# Stylized faces enhance ERP features used for the detection of emotional responses

**DOI:** 10.3389/fnhum.2023.1160800

**Published:** 2023-04-27

**Authors:** Luis Alberto Barradas-Chacón, Clemens Brunner, Selina C. Wriessnegger

**Affiliations:** ^1^Institute of Neural Engineering, Graz University of Technology, Graz, Austria; ^2^Institute of Psychology, University of Graz, Graz, Austria

**Keywords:** EEG, ERP, N170, affect decoding, emotion classification, affective BCI

## Abstract

For their ease of accessibility and low cost, current Brain-Computer Interfaces (BCI) used to detect subjective emotional and affective states rely largely on electroencephalographic (EEG) signals. Public datasets are available for researchers to design models for affect detection from EEG. However, few designs focus on optimally exploiting the nature of the stimulus elicitation to improve accuracy. The RSVP protocol is used in this experiment to present human faces of emotion to 28 participants while EEG was measured. We found that artificially enhanced human faces with exaggerated, cartoonish visual features significantly improve some commonly used neural correlates of emotion as measured by event-related potentials (ERPs). These images elicit an enhanced N170 component, well known to relate to the facial visual encoding process. Our findings suggest that the study of emotion elicitation could exploit consistent, high detail, AI generated stimuli transformations to study the characteristics of electrical brain activity related to visual affective stimuli. Furthermore, this specific result might be useful in the context of affective BCI design, where a higher accuracy in affect decoding from EEG can improve the experience of a user.

## 1. Introduction

Emotions are a complex subjective phenomenon. Many scientific models for studying emotions have been created to work under different contexts. Eckman’s model of basic emotions, for example, defines categorical emotions based on facial expressions common across cultures, such as joy, anger, disgust, surprise, sadness, and contempt (Ekman, 2005). This categorical model of emotions has greatly impacted theories of affective neuroscience, and has led to models that attempt to classify emotions experienced by humans into categories. Another common example of a widely used model of categorical emotions was defined by Robert (1991), based on human and animal behavioral traits. The DEAP dataset, one of the most widely used datasets for emotion detection from electroencephalography (EEG), contains tags for the emotions of pride, elation, joy, satisfaction, relief, hope, interest, surprise, sadness, fear, shame, guilt, envy, disgust, contempt, and anger (Koelstra et al., 2012).

Although categorical models are helpful, when using them to solve the problem of emotion detection from EEG, machine learning engineers rarely attend to the neurophysiological models of emotions. This means that models that accurately predict an emotional category might not have strong corresponding neural correlates (Torres et al., 2020).

To make sense of emotions from a neuroscientific perspective, an approach focused on the human embodied experience of emotion can be used. The most widely accepted theory of emotions in humans, *the theory of constructed emotions*, postulates that there are two factors of an affective response: core affect and emotion (Lewis et al., 2010). In the context of this theory, when an emotional stimulus is presented to a human, emotions do not exist as a categorical neural pattern in the brain, but are rather constructed from core affect. Core affect, as defined by Russell and Barrett (1999), is “the constant stream of transient alterations in an organism’s neurophysiological and somatovisceral state that represent its immediate relationship to the flow of changing events.” That is, affect is the immediate representation of internal states. Affect is commonly represented in two dichotomic variables related to immediate response: valence and arousal. The representation or encoding of affect can later be used by the brain to create or construct a subjective experience, this is considered a constructed emotion. Russell (2003) suggests that core affect can become a constructed emotion through object-directed attributions or appraisals. This definition allows for a complex understanding of emotion that depends on the biological, psychological, and social context of the person experiencing it.

An example to understand the difference of these two definitions is looking at a video of a stranger with a neutral face expression changing to a smiling expression. The immediate change of state in the viewers as measured by neurophysiological variables would be considered a change in core affect. At the same time, the attributions that a person makes about that specific stimulus in the current context determine the constructed emotion. The emotion depends on the individual’s present and past states.

### 1.1. Affect and emotions in the brain

Human faces have been selected as stimuli for this experiment, because they could establish a future connection to models of categorical human emotions (like the Eckman model). Furthermore, brain activity patterns elicited by faces expressing different emotions might be measurable with EEG (Bentin et al., 1996).

A specialized structure in the brain has been described to specifically encode a representation of visual stimuli belonging to faces and face-like images: the Fusiform Face Area (FFA), although varying slightly between individuals, is located in the medial anterior face of the left temporal lobe (Kanwisher et al., 1997; Kanwisher and Yovel, 2006). Using different brain imaging techniques, the FFA has been consistently found to activate about 170 ms after a person perceives a face. Functional magnetic resonance imaging (fMRI) and magnetoencephalography (MEG) were first used to describe the FFA (Halgren et al., 2000). Transcranial magnetic stimulation (TMS) of the FFA also disrupts the perception of facial expressions (Pitcher et al., 2008).

For EEG analysis of the FFA, event-related potentials (ERPs) are used to study the negative electric potential at around 170 ms after a participant has seen a face, the so-called N170. Studies of the N170 interpret a negative potential in the left temporal lobe 170 ms after stimulus onset as the usage of resources on the encoding of faces (Näätänen, 1988; Gao et al., 2019). The N170 has also been found to be a useful feature to reliably classify the affect of stimulus faces, indicating an encoding of core affect in the FFA (Blau et al., 2007).

While affective encoding in the N170 can be used for affect classification, emotion classification has few reliable features that work across different participants and studies. The most reliable features relate to connectivity between frontal and left temporal lobes, as well as inter-hemispheric frontal connectivity. However, a simple interpretation of the theory of constructed emotions is that the construction of an emotional experience requires cognitive resources. Whereas this study aims to measure some of the most common features for emotion classification (frontal late cortical potentials, frontal differential entropy, and N170 peak and mean voltage), a more comprehensive list of features used for emotion selection is available in Zheng et al. (2019).

### 1.2. Faces as visual stimuli

Visual encoding of faces is a widely researched phenomenon. By modifying the properties of the stimulus faces and comparing the resulting activity between the original stimuli and the modified images, inferences can be made about the internal representation of faces. Yang et al. found that the N170 amplitude increases when there are modified face characteristics that make participants struggle with facial recognition. This indicates extra resource allocation for encoding a face when, for example, blocking certain areas of that face (Yang et al., 2020). Liu et al. (2016) found that Chinese opera masks elicited a reduced N170 amplitude as compared to normal faces. Jiang et al. (2016) were able to classify faces with negative vs. neutral emotions from the Chinese Affective Picture System using EEG from a single trial of 1 s. For the detection of emotions from EEG, faces are not a common elicitation method. Studies generally use videos or pictures with a more general theme. In this case, late cortical potentials (LCP) have been found to be relevant for the classification of emotions from EEG (Rahman et al., 2021). Cuthbert et al. (2000) found that emotionally loaded images from the International Affective Picture System (IAPS) produce a larger ERP amplitude from 300 ms and up to 1 s after stimulus onset. A larger frontal LCP suggests extra resource allocation. Common features acquired from frontal EEG for emotion recognition are consistent with the resource allocation required for the construction of emotions (Singh and Singh, 2021).

### 1.3. Modifying visual stimuli for cognitive ease

To explore brain activity related to visual stimuli, images are commonly manipulated by adding or removing visual features (Jiang et al., 2016; Liu et al., 2016). Manipulations that exaggerate the features of a face influence ERP features in an opposite way than manipulations that obstruct or remove visual features of faces (Itz et al., 2016).

For this reason, we hypothesize that a stimulus modification that eases the processing of the visual features of a face can facilitate the emotional processing of the facial expression, thus increasing the emotional response and consequently enhancing the features used for emotion detection from EEG. To measure the processing of said emotions, neural correlates were selected from ERP features.

Although methods vary, studies consistently find evidence for characteristics of EEG related to both affect and emotion. Our objective in this study is thus to find a reproducible experimental design where a consistent modification of the visual features will result in the improvement of the neural correlates used for emotion and affect detection from ERPs. To achieve this, the experimental design must be able to disentangle the features that correlate with affective encoding from those correlating with the construction of emotional experience. Since categorical emotion detection is a difficult problem, many EEG features have been proposed, but few are consistent across experiments. With this objective in mind, two variables for emotion detection have been selected: late frontal mean amplitude and differential entropy (DE).

Late cortical potentials are the amplitude of the EEG signal after a specific time with respect to an event onset compared against a baseline before stimulus onset. In this study, a window of 300–600 ms after the onset was selected. LCPs are commonly interpreted as differences in attention or resource allocation (Näätänen, 1988; Keil et al., 2002; Lang et al., 2013; Zhu et al., 2015).

Differential entropy as a feature for categorical emotion classification has been found to yield high accuracies on emotional classification tasks, but most importantly for this study, it has been found to be a reliable feature across experimental designs (Duan et al., 2013; Torres et al., 2020).

The last two variables were selected to represent emotional encoding. They are selected within the temporal limits for conscious experience, and align with the presented theory as markers for the constructed emotion. Although they might intrinsically also encode the affect that constructed them, they are considered a measure of the resources used to convert the stimulus affect to a meaningful experience of emotion.

N170 Mean and N170 Peak voltages were evaluated as descriptors for the encoding of affect of the stimulus face. The peak was selected automatically, and the mean voltage was calculated in a 160–200 ms window after stimulus onset (Hinojosa et al., 2015; Tian et al., 2018).

## 2. Materials and methods

This study was approved by the ethics committee of the Medical University of Graz and was conducted according to the Declaration of Helsinki. Participants were informed and their consent was obtained before any data collection was done. All participants agreed to the collection and publication of their anonymized data.

### 2.1. Participants

Twenty-eight healthy volunteers (1 left-handed, 14 female, average age 27 ± 4.3 years) participated in this experiment. In the data verification section, the data of two participants was rejected, one male and one female, so EEG analysis was done only with 26 participants. None of the participants reported cognitive, mental, or neurological disorders. All participants had normal (or corrected) vision. Before the measurement, participants reported their perceived affective state and traits with the State and Trait Anxiety and Depression Inventory (STADI). No participant scored above 0.5 of the normalized scores for the different scales of the STADI. This is interpreted as none of the participants having mild, moderate, or severe depression or anxiety in neither trait nor state evaluations.

### 2.2. Signal recording

Signals were recorded during the visual presentation phase of the experiment. Participants sat comfortably on an office chair inside a dimly lit, acoustically dampened, and electromagnetically shielded booth. They were asked to maintain central fixation and minimize blinks and movements throughout the measurement. The stimuli presentation consisted of three blocks lasting 12 min each. Participants were given time to rest between blocks. The total duration of the experimental session did not exceed 1 h for any participant.

### 2.3. Experimental paradigm

**FIGURE 1:**
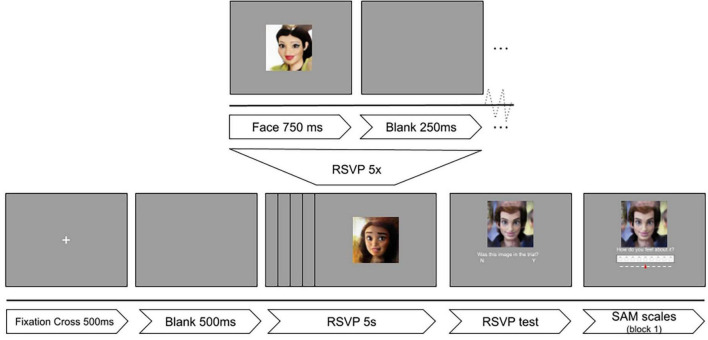
Experimental timeline of the RSVP protocol. Reproduced with permission from Mollahosseini et al. (2017), available at mohammadmahoor.com/affectnet/.

For the presentation of the images, a modification of the rapid serial visual presentation (RSVP) protocol (Ying and Xu, 2016) was implemented in PsychoPy v2021.1.4 (Peirce et al., 2019). Images were shown to participants on a 24 inch monitor (61 cm diagonal at a resolution of 1,920 × 1,080 pixels). Each of the three blocks consisted of 300 images (that is, 100 images in three categories). The flow diagram for the experimental design can be seen in Figure 1.

Individual trials consisted of rapid serial visual presentation of five images. After every presentation of five images, a sixth face and a question was shown: “Was this face in this trial?” Participants had to respond if the sixth image was presented among the previous five faces. The correct answer to this question was “Yes” in half of the trials. The goal of this question was to keep participants’ attention and motivation high, as well as gamifying the experiment by providing a final score. For the first block of the experiment, participants were also asked to rate the image based on the Self-Assessment Manikin (SAM) scales for valence and arousal (Peacock and Wong, 1990).

#### 2.3.1. Stimulus material

One hundred images were selected from the AffectNet dataset (Mollahosseini et al., 2017) using the following criteria: frontal or mostly frontal human faces of all ages in full color without accessories or partially covered. The selected images were obtained by iterative random sampling and visually filtering out images with undesired characteristics. Images smaller than the desired resolution were excluded, and images with a larger resolution were resized to half the height of the display resolution (540 × 540 pixels). The images were selected from a pool of 103,479 faces from the AffectNet dataset, hand-labeled for Valence and Arousal. They were sampled randomly from this pool to obtain a uniform distribution of affect. To do this, a cartesian plane with boundaries at −1 and 1 was used from two affect variables: valence and arousal. A circle of radius 0.5, and center at (0, 0) was used for a neutral category. Four categories were then created for each of the cartesian planes. This created a total of 20 pictures for each of 5 affect categories. After this process, a human-driven selection of undesired images was executed: faces that were partially covered, had too much makeup, or were visually unclear were replaced with images with the same facial expression of emotion. Unfortunately, the reported affect of the new images was not controlled for. This yielded a non-uniform distribution of the affect quartiles.

#### 2.3.2. Image modifications

**FIGURE 2:**
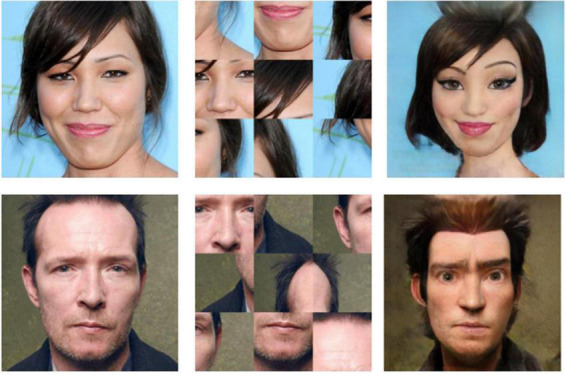
Example of the three image transformations that compose the three categories of stimuli: IMG (Left), SF (Center), and TOON (Right). Reproduced with permission from Mollahosseini et al. (2017), available at mohammadmahoor.com/affectnet/.

Two modifications were applied independently to every image to create three experimental categories: control, scrambled, and cartoonish faces. The control category is referred to as IMG. The category of stimuli for scrambled faces (SF) was created by splitting every image in the control group into a 3 × 3 grid and randomly shuffling the position of the tiles, producing the effect of scrambling the visual features of the face. The cartoonified (TOON) category of stimuli was created by subjecting the images in the control group to a filter based on DL style transfer. This style transfer model was trained by Pinkney and Adler (2020) to transfer the style of cartoon characters onto photos of human faces. The model is not publicly available, but an API can be used to apply the neural filter on relatively small images. The filter exaggerates features that animators use to express emotion, which includes increasing the size of the eyes and mouth, but also changing details like blushing, adding makeup, or softening textures. Examples of the modifications are provided in Figure 2.

### 2.4. EEG recording and processing

**FIGURE 3:**
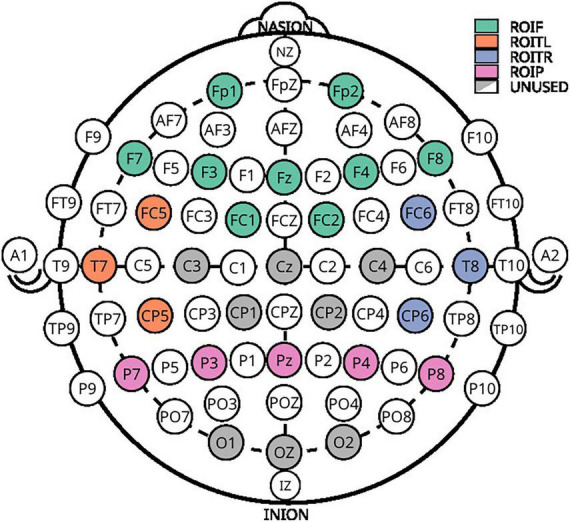
Electroencephalography layout used for acquisition. Colored electrodes represent the selected positions from the extended 10–20 system.

Electroencephalography was recorded from 28 locations with a 32 channel LiveAmp amplifier (Brain Products GmbH, Germany) at a sampling rate of 500 Hz. All EEG impedances were below 25 kΩ. Four additional channels were used to record EOG. The recorded locations of the extended 10–20 system are Fp1, Fz, F3, F7, FC5, FC1, Cz, C3, T7, CP5, CP1, P3, P7, Pz, O1, Oz, O2, P4, P8, CP6, CP2, C4, T8, FC6, FC2, F4, F8, and Fp2, as seen in Figure 3.

Collection was done with Brain Products’ BrainVision Recorder Software synchronized *via* LSL with stimulus markers from the stimuli paradigm. The recordings of subjects 14 and 22 were rejected due to missing stream data in the XDF file. Biosignals were processed with MNE-Python v0.24.1 (Gramfort et al., 2013). Bad channels for every participant were selected through visual inspection. These were substituted by interpolating neighboring channels. The data was filtered (FIR, hamming window, bandpass 0.1–30 Hz), re-referenced against a common average (CAR), and epoched with a window of (−200 to 800 ms) relative to the onset of the face presentation. A baseline correction of 200 ms before the stimulus onset was used. Trials with a peak-to-peak amplitude (V_p–p_) larger than 80 μV for EEG channels were automatically rejected, as well as trials of EOG V_p–p_ larger than 1 mV. Trials with channels that had less than 0.1 μV as a minimum signal V_p–p_ were considered flat and rejected. No recording presented more than 10% of trial rejection.

### 2.5. Feature extraction

The following ERP-related features were extracted from the EEG signals.

#### 2.5.1. Absolute mean amplitude

The absolute mean amplitude is the average value of a given signal in a 300–600 ms window over the frontal region electrodes. This variable has been reported to modulate emotional processing. It is a frontal cortical potential that is assumed to relate to the amount of cognitive resources used for the construction of an emotional experience.

#### 2.5.2. Differential entropy

The signal’s differential entropy in the specified window is defined as:


$$h\,\left(x\right)=-\int_{-\infty }^{\infty }\frac{1}{\sqrt{2\,\pi \,\sigma^{2}}}\,e^{\frac{{\left(x-\mu \right)}^{2}}{2\,\sigma^{2}}}\cdot l\,o\,g\,\left(\frac{1}{\sqrt{2\,\pi \,\sigma^{2}}}\,e^{\frac{{\left(x-\mu \right)}^{2}}{2\,\sigma^{2}}}\right)\,dx$$

$$=\frac{1}{2}\,l\,o\,g\,\left(2\,\pi \,\sigma^{2}\right)$$

where *x* is the EEG signal, assumed to have a normal distribution *N*(μ,σ^2^). This variable has been found to consistently produce high machine learning accuracies for affect detection.

#### 2.5.3. N170 mean and peak amplitude

When analyzing the temporal left region of interest, in the window of 160–200 ms, the mean amplitude of the EEG signal relates to the average amount of resources used for the cognitive encoding of specific faces. The largest negative peak was also used as a descriptive feature of the N170.

## 3. Results

**FIGURE 4:**
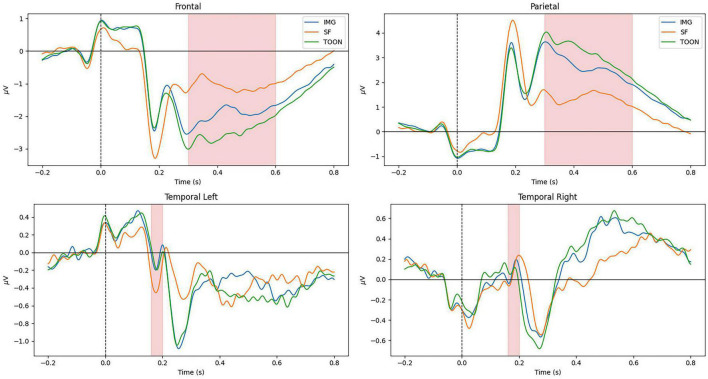
Grand average ERP on frontal, parietal, temporal left, and temporal right regions of interest aggregated by experimental categories: control (IMG), scrambled (SF), and cartoonish (TOON) faces.

All participants scored below 0.5 of the normalized scores for the different scales of the STADI, meaning no participant presented neither moderate nor severe depression or anxiety scores. Figure 4 shows the average ERP in the frontal (Fz, Fp1, F3, F7, FC1, FC2, F4, F8, and Fp2), parietal (Pz, P3, P7, P4, and P8), temporal left (FC5, T7, and CP5), and temporal right (FC6, T8, and CP6) ROIs across all participants aggregated by experimental conditions. The marked windows are: 300–600 ms for frontal and parietal, and 160–200 ms for temporal ROIs.

Late cortical potentials present a significant difference between the three experimental conditions, where the SF condition has the smallest amplitude, followed by the IMG and TOON conditions. Analysis of variance was used for the comparison of these differences for each variable. Repeated measures ANOVAs were performed with *condition* (3) × *sex* (2) as within- and between-subject variables. An alpha value of 0.05 was selected for all statistical tests, and presented results were adjusted using Tukey’s correction for multiple comparisons. Results from these analyses can be observed in Table 1.

**TABLE 1:** Results from ANOVA tests.

	*F*(2,48)	*p*-Value	η^2^
LCP	65.812	<0.001	0.245
DE	31.15	<0.001	0.052
N170 peak	3.384	<0.05	0.02
N170 mean	3.14	0.052	0.018

The difference in frontal LCP between conditions was found significant [*F*(2,48) = 65.812, *p* < 0.001, η^2^ 0.245]. *Post-hoc* tests revealed all conditions to be significantly different [IMG-SF: *t*(24) = −5.91, *p* < 0.001; IMG-TOON: *t*(24) = 6.76, *p* < 0.001; SF-TOON: *t*(24) = 10.36, *p* < 0.001].

Results were also confirmed when looking at the DE variable: a significant difference between conditions was found [*F*(2,48) = 31.15, *p* < 0.001,η^2^ = 0.052]. *Post-hoc* tests for the same analysis revealed significant differences between the SF condition and the rest [IMG-SF: *t*(24) = −6.10, *p* < 0.001; SF-TOON: *t*(24) = 5.44, *p* < 0.001], but not between IMG and TOON [IMG-TOON: *t*(24) = 1.67, *p* = 0.237]. The relevant post-hoc tests can be observed in Table 2. No significant difference between sexes was found.

**TABLE 2:** *Post-hoc* results for variables LCP and DE.

	IMG-SF *t*(24)	IMG-TOON *t*(24)	SF-TOON *t*(24)
LCP	−5.91, *p* < 0.001	6.76, *p* < 0.001	10.36, *p* < 0.001
DE	−6.10, *p* < 0.001	1.67, *p* = 0.237	5.44, *p* < 0.001

**FIGURE 5:**
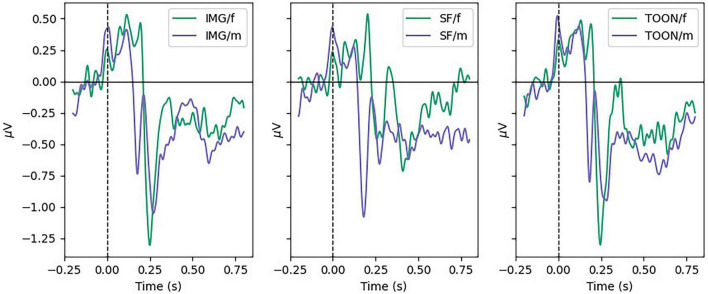
Grand average ERP in temporal left ROI for male and female participants in each condition.

Figure 5 shows the average ERP for all participants, aggregated by sex, between conditions in the Temporal Left ROI (FC5, T7, and CP5). A difference in N170 Peak amplitude was shown to be significant with the help of a repeated-measures ANOVA [*F*(2,48) = 3.384, *p* < 0.05, η^2^ 0.020], but *post-hoc* tests showed no significant differences within conditions. Instead a significant difference was found in the between subject factor *sex* [*F*(2,48) = 11.6, *p* = 0.002, η^2^ 0.090]. No significant differences were found for the N170 Mean variable [*F*(2,48) = 3.140, *p* = 0.052, η^2^ 0.018].

### 3.1. Differences in N170 between sexes

**FIGURE 6:**
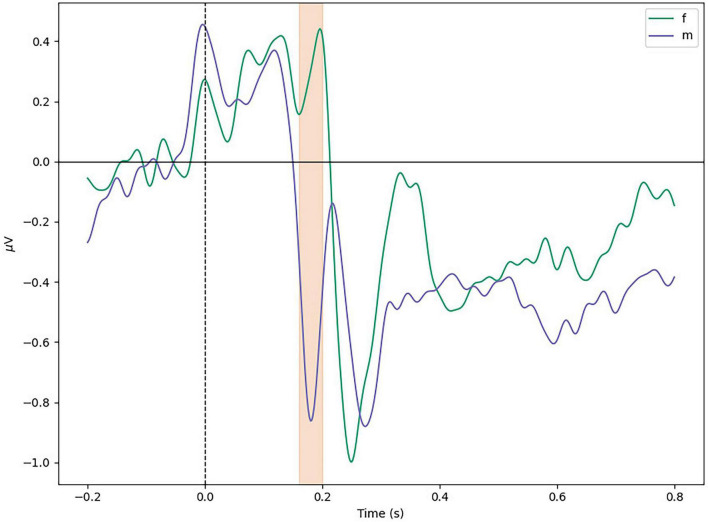
Average difference between sexes in temporal left ROI. Marked window is 160–200 ms.

As mentioned in the last section, and demonstrated in Figure 6, women present a smaller N170 peak. This difference was found significant by *post-hoc* tests (*t* = 3.41, *p* = 0.002).

Rapid serial visual presentation task accuracy was measured as the number of faces correctly remembered to either belong or not in the presented RSVP trial. RSVP task accuracy presented a small [*m* = 158.42 (9.64), *f* = 166.36 (5.59), *d* = 0.96] but significant difference between sexes. This difference was found significant with the help of a Mann–Whitney test (*U* = 48.5, *p* = 0.024). These results suggest that the visual processing of faces might be done differently between sexes.

## 4. Discussion

We hypothesized that by easing the visual processing of an image we could enlarge features used for affect and emotion detection from EEG. We found that LCP and DE variables significantly improve in the TOON category when compared to IMG and SF, showing that the selected biomarkers for emotion and affect improve with the TOON category.

Variables N170 Mean and N170 Peak showed no significant difference in the within-subject factor *category*, but showed a significant difference in the between-subject factor *sex*. Female participants present on average a significantly earlier and smaller N170 ERP. At the same time, RSVP score results were significantly better for female participants. This difference in the N170 of participants of different sex could be the reason why no significant results in the affect variables were found. Previous studies have shown that “women consistently perform better in face memory tasks than men and also show earlier and larger N170 components” (Nowparast Rostami et al., 2020). Our experiment shows earlier, but smaller N170 peaks in female participants, but also significantly better scores at the RSVP task. We can thus interpret that women in our task were allocating significantly less resources than men for face encoding and processing, while at the same time performing significantly better at the given task. This finding suggests models for affect classification that have bases in the N170 peak can improve their results by integrating information about the participant’s sex. This is a factor that, to our knowledge, current classification benchmarks do not control for Choi et al. (2015).

An assumption is being made, considering that the N170 amplitude is related to the RSVP performance score. This score has to do with correct answers in the memory task, which does not necessarily reflect participant’s perceived difficulty. Future experimental designs could include a perceived task difficulty questionnaire to confirm or deny these results. Lastly, if female participants are not requiring as much resources for the task, its difficulty could be raised, by increasing the number of faces to remember, for example, to explore if the RSVP score and the N170 amplitude relate in the proposed way.

The results from this study seem to indicate a visual stimulus that can significantly modify emotional processing, as measured by LCP and DE, without modifying the affective encoding of the observed faces as measured by the N170. However, good results have already been obtained for affect classification from observed objects using only information from the N170 and relatively simple linear models. The current accuracy score for binary classification of Negative vs. Positive valence is 83% with LDA, 84% with SVM, and 86% with Lasso, achieved by Tian et al. (2018). These models achieve a high accuracy without information from the observer’s sex. Being linear, they are not best suited for modeling conditioning variables, like age, personality, or handedness.

Models that integrate the information of the participant’s sex could improve the current accuracy benchmark in affective detection. An interaction between the sex of the participant and the sex of the person on the image has not yet been researched, and could provide yet another insight about the neural correlates of affect and emotion.

Many other features of the human EEG can convey information about affect encoding and emotional processing. Connectivity measures have proved to be useful when classifying affective states (Costa et al., 2006; Lee and Hsieh, 2014). Integrating these models could provide additional information about the process of emotional construction from affective encoding.

## 5. Conclusion

In this study we showed how, by means of a style transfer image modification, images of human faces influenced the ERP features used for emotion detection. Features used for affect classification were not significantly modified. This demonstrates a method that can disentangle affective encoding from emotional processing, by exploiting specific neural correlates to core affect, and constructed emotions. We attribute the effect to the visual ease of processing provided by the TOON category.

The significant difference between sexes in N170 ERPs suggests that models can benefit from being informed about the observer’s sex.

These findings enable the study of different neural correlates of emotion elicitation, while providing a reproducible method to enhance the emotional response using DL style transfer. We envision this specific result to be useful in the context of affective Brain-Computer Interfaces (BCI) design, where a higher classification accuracy in emotion and affect detection from EEG can improve the experience of a user.

A relevant future contribution is the dataset created through this experiment. Currently, in the task of emotion classification from EEG, there exist only two standard datasets. The publication of the data collected in this project will provide a machine learning benchmark that diversifies the definition of the current task of emotion detection from EEG.

## Data availability statement

The datasets presented in this study can be found in online repositories. The names of the repository/repositories and accession number(s) can be found below: https://osf.io/8knau.

## Ethics statement

The studies involving human participants were reviewed and approved by the Medical University Graz. The patients/participants provided their written informed consent to participate in this study.

## Author contributions

LB-C and SW conceived the experiments. LB-C conducted the experiments and analyzed the results. LB-C, CB, and SW reviewed the manuscript. All authors contributed to the article and approved the submitted version.
